# An Inquiry Concerning the Ava Root

**Published:** 1813-02

**Authors:** 

**Affiliations:** London


*To the Editors of the Medical and Physical Journal.*

GENTLEMEN,

GENTLEMEN,

IN Captain Dixon's Voyage round the World, frequent mention is made of the intoxicating and injurious effects of a root called Ava. It is described to be a root resembling liquorice in shape and color, but not in taste. It is first chewed, then put in a wooden bowl, in which a small quantity of water is poured upon it; the liquor is afterwards strained through a cloth, and drank by the higher ranks, (for none but *arees* or chiefs are permitted to taste the delicious beverage,) in some of the South-Sea islands.

Besides the intoxicating effects of this mixture, which however seems rather to stupify than to exhilarate, its long-continued use is stated to occasion emaciation, and an appearance on the skin resembling white scurf and leprosy. As this account is not given by a medical man, I should be glad to learn, from some of your ingenious correspondents, a more particular history of this plant, which seems to claim a place among the narcotics; also some more accurate detail of its effects on the human system.

I am, Gentlemen, &c. &c.

*London, Jan.* 5, 1813. INDAGATOR.

